# Anemia in a cohort of HIV-infected Hispanics: prevalence, associated factors and impact on one-year mortality

**DOI:** 10.1186/1756-0500-7-439

**Published:** 2014-07-08

**Authors:** Eduardo J Santiago-Rodríguez, Angel M Mayor, Diana M Fernández-Santos, Yelitza Ruiz-Candelaria, Robert F Hunter-Mellado

**Affiliations:** 1 Retrovirus Research Center, Universidad Central del Caribe School of Medicine, 00960-6032 Bayamón, Puerto Rico; 2 Cancer Research Center, Universidad Central del Caribe School of Medicine, Bayamón, 00960-6032 Puerto Rico

**Keywords:** Anemia, HIV, Hispanics, Prevalence, Mortality, Puerto Rico

## Abstract

**Background:**

Anemia occurs frequently in HIV-infected patients and has been associated with an increased risk of death in this population. For Hispanic subjects, information describing this blood disorder during HIV is scarce. Therefore, the present study examined data from a cohort of HIV-positive Hispanics to determine the prevalence of anemia, identify its associated factors, and evaluate its relationship with one-year mortality.

**Methods:**

This study included 1,486 patients who enrolled between January, 2000 and December, 2010 in an HIV-cohort in Bayamón, Puerto Rico. Data were collected through personal interviews and medical record abstractions. To determine the factors independently associated with anemia, a multivariable logistic regression model was used. Kaplan-Meier and Cox proportional hazards models were also performed to estimate survival time and to predict death risk.

**Results:**

The prevalence of anemia at enrollment was 41.5%. Factors independently associated with increased odds of anemia were: unemployment (OR = 2.02; 95% CI 1.45-2.79), CD4 count <200 cells/μL (OR = 2.66; 95% CI 1.94-3.66), HIV viral load ≥100,000 copies/mL (OR = 1.94; 95% CI 1.36-2.78), white blood cell count <4,000 cells/μL (OR = 2.42; 95% CI 1.78-3.28) and having clinical AIDS (OR = 2.39; 95% CI 1.39-4.09). Overweight (OR = 0.43; 95% CI 0.32-0.59) and obese (OR = 0.44; 95% CI 0.29-0.67) BMI’s were independently associated with reduced odds of anemia. Survival differed significantly by anemia status (log-rank test: p < 0.001). One-year mortality estimates were: 30.8%, 23.3%, 8.4% and 2.5%, for patients with severe, moderate, mild and no anemia, respectively. Having anemia at baseline was independently associated with an increased one-year mortality risk (severe anemia: HR = 9.06; 95% CI: 4.16-19.72; moderate anemia: HR = 6.51; 95% CI: 3.25-13.06; mild anemia: HR = 2.53; 95% CI: 1.35-4.74).

**Conclusions:**

A high prevalence of anemia at enrollment was observed in this cohort of HIV-infected Hispanics. Unemployment and several adverse prognostic features of HIV infection were independently associated with this blood disorder. Anemia resulted to be the strongest predictor of one-year mortality, evidencing a dose–response effect. Further investigations are needed to evaluate whether recovering from anemia is associated with longer survival, and to identify the types of anemia affecting this particular group of HIV patients.

## Background

The development of hematological disorders in HIV-infected patients was described a few years after the first cluster of AIDS cases was identified [1,2]. Usually defined as low hemoglobin levels or low hematocrit, anemia is the most common cytopenia seen in the course of HIV infection [3]. The prevalence of anemia in persons with HIV has been determined in many studies [4-14], with values that range between 19% and 69% [9,10]. Besides its commonness in people having HIV, there has been recognized a linkage between anemia and decreased survival in this population [15-18]. In fact, anemia has been associated with an increased risk of death in HIV-infected patients, independently of many indicators of poor prognosis, such as: low CD4 cell count, high HIV viral load or the manifestation of AIDS-defining conditions [4,7,12,19-21].

The mechanisms associated to anemia in the context of HIV infection are broadly classified in relation to an inefficient hematopoietic process, resulting from: malnutrition, co-infections, neoplasms, decreased erythropoietin production and the use of antiretroviral medications [3,15,22]. Additional mechanisms are related to an increased red blood cell (RBC) lost or destruction produced by gastrointestinal or genitourinary bleeding, and entrapment of RBC in the spleen [3,22]. There are also elements of socio-demographic, immunological and clinical nature that have been frequently associated to the coexistence of anemia in the HIV-infected population. Some of these factors include: female sex [5,9,10,19], increasing age [7,8], high HIV viral load [6,8,20], low CD4 cell count [5-10,12-14,20], presenting opportunistic infections (clinical AIDS) [5,6,20], low body mass index (BMI) [7,10,14] and intravenous drug use [5]. Other cytopenias, including leucopenia and thrombocytopenia, have been also associated to anemia in HIV-positive patients [19].

Although information describing anemia during HIV is already published, limited data is available on the prevalence and impact of this disorder in specific patient groups [16], as it is with the Hispanic population. Hispanics have been disproportionately affected by the HIV epidemic [23]. In 2010, they comprised 16% of the total population living in the United States and its territories [24], but accounted for the 21% of the new HIV cases reported [25]. The present study examined data from a cohort of HIV-infected patients living in Puerto Rico, a commonwealth territory of the United States in which 99% of its inhabitants are Hispanics [24]. The objectives of this study were directed to: 1) determine the prevalence of anemia, 2) identify its associated factors and 3) evaluate the relationship between this hematological disorder and one-year mortality.

## Methods

### Setting and study population

This study included HIV-positive adults (21 years or older) who received their HIV-associated health care in the Universidad Central del Caribe academic health center (UCC) in Bayamón, Puerto Rico, either at the Ramón Ruiz-Arnau University Hospital or at the HIV ambulatory clinics. All patients with documented HIV infection seen in these facilities are invited to enroll in the Retrovirus Research Center HIV-cohort. If the patient agrees to participate, the aims and procedures of the study are explained and a consent form is read, discussed and upon acceptance, it is signed by the participant. Then, face to face interviews and blood sample collections are performed. After this initial assessment subsequent visits are scheduled every six months. Patients considered for this study had their enrollment between January, 2000 and December, 2010. The study was approved by the UCC’s Institutional Review Board.

### Data collection and variables of interest

In this study we evaluated baseline information only. The information collected at the first visit covered the period within the date of interview and its preceding year. In addition to personal interviews, medical record abstractions were used as data collection methods. The laboratory results obtained from the blood drawn during the day of visit were incorporated to the patients’ records before abstraction. For patients in which multiple laboratory or clinical measurements were available, only the most recent ones were considered in the analysis.

The variables examined in this study were: age (≤35 years, 36–45 years, >45 years), sex (male, female), level of education completed (less than high school, high school, college), employment status (employed, unemployed), lifetime alcohol use (yes, no), lifetime intravenous drug use (yes, no), CD4 cell count (<200 cells/μL, ≥200 cells/μL), HIV viral load (<10,000 copies/mL, 10,000-100,000 copies/mL, >100,000 copies/mL), platelets count (<150,000 cells/μL, ≥150,000 cells/μL), white blood cell count (<4,000 cells/μL, ≥4,000 cells/μL), clinical AIDS (yes, no) and antiretroviral treatment use (yes, no). BMI was estimated using patients’ weight and height employing the following formula: weight (kg) / [height (m)]^2^. Patients’ BMI was further classified as underweight: <18.5 kg/m^2^, normal: 18.5-24.9 kg/m^2^, overweight: 25.0-29.9 kg/m^2^ and obese: ≥30.0 kg/m^2^. Finally, anemia was defined as hemoglobin levels < 12 g/dL in women and < 13 g/dL in men, according to established guidelines [26].

For the survival analysis, as per a cooperative agreement with the Puerto Rico Department of Health, mortality information was obtained from their mortality registry, up to December, 2011. Patients who did not have mortality data in the first year after study enrollment were considered as censored. In this analysis, anemia status was sub-classified as: severe anemia (<8 g/dL), moderate anemia (8–9.9 g/dL) and mild anemia (10–11.9 g/dL in women and 10–12.9 g/dL in men).

### Statistical analysis

Descriptive statistics were used to illustrate the characteristics of the population under study and the overall prevalence of anemia at enrollment. Prevalence of anemia by socio-demographic, immunological and clinical characteristics were also calculated and compared through Chi-square tests. Variables with statistically significant differences on that bivariate analysis were further evaluated in a multivariable model to determine the factors independently associated with anemia. Logistic regression was employed and results were reported as odds ratios (OR) with their respective 95% confidence intervals (95% CI). Overall fit of the model was measured with the Hosmer-Lemeshow goodness of fit test and ascertained (p = 0.830).

Kaplan-Meier technique was also performed to estimate one-year survival according to anemia status at enrollment. Differences in survival time were measured with the log-rank test. Finally, a Cox proportional hazards model was executed to assess the association between baseline anemia and risk of dying after one year, controlling for possible confounders. Age, sex, BMI, CD4 cell count, HIV viral load, clinical AIDS and antiretroviral treatment, all previously associated with mortality in HIV-infected patients [4,7,20,21,27], were included in the final model after an initial evaluation using univariable analyses. Results were reported as hazard ratios (HR) with corresponding 95% CI’s. The statistical software Stata/SE (Version 12.1, College Station, TX, USA) was used to carry out the analyses. All tests were two-sided and significance was set at 0.05.

## Results

### Characteristics of the population studied and prevalence of anemia

Between January, 2000 and December, 2010 a total of 1,502 patients were enrolled in the cohort. Of them, 16 patients did not have hemoglobin measurements at enrollment and were excluded of the study, for a total of 1,486 subjects. Median age of study participants was 40 years (range, 21–79), 67.3% were male, 68.6% had an education level of at least high school and 25.0% were employed. More than half of participants (51.5%) used alcohol and 34.4% used intravenous drugs. A total of 41.8% had a CD4 cell count <200 cells/μL, 9.5% presented clinical AIDS and 64.4% received antiretroviral medications. The overall prevalence of anemia at enrollment was 41.5% (Table 1).

**Table 1:** Description of study participants (n = 1,486)

**Characteristic**	**%**
Males	67.3
Age, median (range)	40 (21–79)
Education level*	
Less than high school	31.4
High school	39.2
College	29.4
Employment status*	
Employed	25.0
Unemployed	75.0
Alcohol use*	51.5
Intravenous drug use*	34.4
Clinical AIDS	9.5
CD4 count <200 cells/μL*	41.8
Received antiretroviral treatment	64.4
Anemia: Hb < 12 g/dL women, < 13 g/dL men	41.5

*Variable had missing values: Education level = 7; Employment status = 25; Alcohol use = 7; Intravenous drug use = 2; CD4 count = 33.

Hb, hemoglobin level.

### Factors associated with anemia

The prevalence of anemia differed significantly (p < 0.05) by: age, education level, employment status, BMI, CD4 cell count, HIV viral load, platelets count, white blood cell count, clinical AIDS and antiretroviral treatment use (Table 2). Anemia was more prevalent among older (46.7% >45 years, 40.8% 36–45 years and 37.4% ≤35 years), less educated (46.9% less than high school, 43.9% high school and 32.0% college) and unemployed (46.6%, 25.5% employed) patients. The presence of anemia was also more frequent among patients with: lower BMI (74.4% underweight, 49.4% normal, 26.8% overweight and 22.7% obese), lower CD4 cell count (63.2% <200 cells/μL, 23.9% ≥200 cells/μL), higher HIV viral load (61.1% >100,000 copies/mL, 34.0% 10,000-100,000 copies/mL and 25.4% <10,000 copies/mL), lower platelets count (56.9% <150,000 cells/μL, 36.7% ≥ 150,000 cells/μL), lower white blood cell count (66.9% <4,000 cells/μL, 29.4% ≥4,000 cells/μL), clinical AIDS (78.7%, 37.6% no clinical AIDS) and antiretroviral medications received (43.4%, 38.0% no ART).

**Table 2:** Relationship between socio-demographic, immunological and clinical characteristics of patients and anemia

	**Prevalence of anemia**		**Univariable analysis**		Multivariable analysis*	
**Variable**	**%**	**p-value**	**OR**	**95% CI**	**OR**	**95% CI**
Sex						
Male	40.0	0.103	1		1	
Female	44.4		1.20	0.96-1.49	1.30	0.98-1.74
Age (years)						
≤35	37.4	0.016	1		1	
36-45	40.8		1.15	0.90-1.48	0.96	0.70-1.32
>45	46.7		1.47	1.13-1.92	1.11	0.79-1.57
Education level						
College	32.0	<0.001	1		1	
High school	43.9		1.66	1.28-2.16	1.32	0.95-1.83
Less than high school	46.9		1.88	1.43-2.47	1.33	0.93-1.89
Employment status						
Employed	25.5	<0.001	1		1	
Unemployed	46.6		2.55	1.96-3.33	2.02	1.45-2.79
Alcohol use						
No	39.5	0.148	1			
Yes	43.2		1.17	0.95-1.43		
Intravenous drug use						
No	41.0	0.658	1			
Yes	42.2		1.05	0.85-1.31		
BMI (kg/m^2^)						
Normal (18.5-24.9)	49.4	<0.001	1		1	
Underweight (<18.5)	74.4		2.99	1.96-4.54	1.50	0.91-2.47
Overweight (25.0-29.9)	26.8		0.38	0.29-0.49	0.43	0.32-0.59
Obese (≥30.0)	22.7		0.30	0.21-0.43	0.44	0.29-0.67
CD4 count (cells/μL)						
≥200	23.9	<0.001	1		1	
<200	63.2		5.46	4.34-6.86	2.66	1.94-3.66
HIV viral load (copies/mL)						
<10,000	25.4	<0.001	1		1	
10,000-100,000	34.0		1.51	1.14-2.01	1.13	0.80-1.59
>100,000	61.1		4.62	3.53-6.03	1.94	1.36-2.78
Platelets count (cells/μL)						
≥150,000	36.7	<0.001	1		1	
<150,000	56.9		2.28	1.78-2.91	1.24	0.89-1.71
White blood cell count (cells/μL)						
≥4,000	29.4	<0.001	1		1	
<4,000	66.9		4.84	3.83-6.12	2.42	1.78-3.28
Clinical AIDS						
No	37.6	<0.001	1		1	
Yes	78.7		6.15	4.05-9.35	2.39	1.39-4.09
Received ART						
No	38.0	0.044	1		1	
Yes	43.4		1.25	1.01-1.55	0.76	0.56-1.03

*Logistic regression, n = 1,307. Some patients (n = 179) were not considered in this analysis because they had missing values in any of the variables included in the model: Education level = 7; Employment status = 25; Alcohol use = 7; Intravenous drug use = 2; BMI = 115; CD4 count = 33; HIV viral load = 27; Platelets count = 9.

BMI, body mass index; ART, antiretroviral treatment; OR, odds ratio; CI, confidence interval.

When stratified by alcohol and intravenous drug use, no significant differences were found in the prevalence of anemia. Therefore, the association between anemia and these variables were not considered beyond this point. All other factors with significant differences in the prevalence of anemia were included in the multivariable model. Sex was not significantly associated to anemia in the bivariate analysis but considering findings from other studies [5,9,10,19], it was also included in the final model.

The results of the multivariable logistic regression model are also presented in Table 2. Unemployment (OR = 2.02; 95% CI 1.45-2.79), CD4 count <200 cells/μL (OR = 2.66; 95% CI 1.94-3.66), HIV viral load ≥100,000 copies/mL (OR = 1.94; 95% CI 1.36-2.78), white blood cell count <4,000 cells/μL (OR = 2.42; 95% CI 1.78-3.28) and having at least one AIDS- defining condition (OR = 2.39; 95% CI 1.39-4.09) were all significantly associated with increased odds of anemia. On the contrary, overweight (OR = 0.43; 95% CI 0.32-0.59) and obese (OR = 0.44; 95% CI 0.29-0.67) BMI’s, compared to normal BMI, were significantly associated with decreased odds of anemia. Sex, age, education level, platelets count and antiretroviral treatment were not associated with the presence of anemia in this model.

### Survival analysis

**Figure 1:**
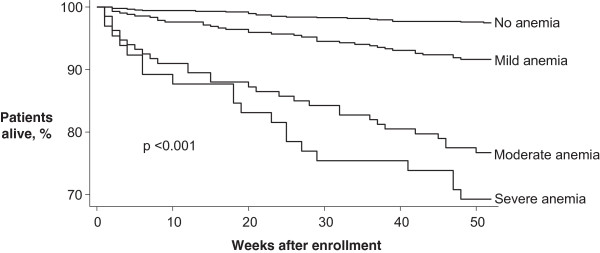
**Kaplan-Meier survival estimates according to anemia status at baseline.** Anemia status based on hemoglobin levels at study entry: severe anemia (<8.0 g/dL), moderate anemia (8.0-9.9 g/dL), mild anemia (10.0-11.9 g/dL in women and 10.0-12.9 g/dL in men) and no anemia (≥12.0 g/dL in women and ≥13.0 g/dL in men). Log-rank test was used to compare survival estimates.

Based on the Kaplan-Meier method, survival at one year differed significantly by anemia status at baseline (log-rank test: p < 0.001, Figure 1). The proportion of patients to have died after a year of follow-up was estimated in: 30.8% for patients with severe anemia, 23.3% for patients with moderate anemia, 8.4% for patients with mild anemia and 2.5% for patients without anemia. The Cox proportional hazards model revealed that after controlling for age, sex, BMI, CD4 cell count, HIV viral load, clinical AIDS, and antiretroviral treatment, having anemia at enrollment increased significantly the one-year mortality risk of these HIV-infected patients. Moreover, the strength of this association was related with the degree of anemia (severe anemia: HR = 9.06; 95% CI: 4.16-19.72; moderate anemia: HR = 6.51; 95% CI: 3.25-13.06; mild anemia: HR = 2.53; 95% CI: 1.35-4.74). Other factors independently associated with one-year mortality were: male sex (HR = 1.73; 95% CI: 1.06-2.81), overweight BMI (HR = 0.34; 95% CI: 0.15-0.76) and antiretroviral treatment use (HR = 0.51; 95% CI: 0.33-0.80) (Table 3).

**Table 3:** Relationship between baseline characteristics and one-year mortality*

	**Univariable analysis**		**Multivariable analysis**^**^**^	
**Variable**	**HR**	**95% CI**	**HR**	**95% CI**
Sex				
Female	1		1	
Male	1.41	0.91-2.17	1.73	1.06-2.81
Age (years)				
≤35	1		1	
36-45	1.64	0.99-2.72	1.05	0.60-1.82
>45	1.91	1.14-3.23	1.41	0.80-2.48
BMI (kg/m^2^)				
Normal (18.5-24.9)	1		1	
Underweight (<18.5)	2.05	1.27-3.29	1.12	0.64-1.96
Overweight (25.0-29.9)	0.21	0.10-0.44	0.34	0.15-0.76
Obese (≥30.0)	0.41	0.20-0.86	0.91	0.42-2.00
CD4 count (cells/μL)				
≥200	1		1	
<200	3.35	2.18-5.15	1.20	0.68-2.09
HIV viral load (copies/mL)				
<10,000	1		1	
10,000-100,000	1.95	1.02-3.69	1.21	0.62-2.37
>100,000	4.04	2.29-7.10	1.55	0.81-2.98
Clinical AIDS				
No	1		1	
Yes	3.64	2.37-5.60	1.17	0.68-2.02
Received ART				
No	1		1	
Yes	0.65	0.45-0.95	0.51	0.33-0.80
Anemia: Hb (g/dL)				
No (≥12.0 women, ≥13.0 men)	1		1	
Mild (10.0-11.9 women, 10.0-12.9 men)	3.41	2.00-5.80	2.53	1.35-4.74
Moderate (8.0-9.9)	10.41	6.03-17.98	6.51	3.25-13.06
Severe (<8.0)	14.40	7.86-26.38	9.06	4.16-19.72

*Cox proportional hazards regression.

^^^n = 1,333. Excluded patients (n = 153) had missing values in any of the variables included in the model: BMI = 115; CD4 count = 33; HIV viral load = 27.

BMI, body mass index; ART, antiretroviral treatment; Hb, hemoglobin level; HR, hazard ratio; CI, confidence interval.

## Discussion

The prevalence of anemia found in our cohort is within the range observed in other studies targeting HIV-infected patients [4-14]. However, when compared to studies in which data on Hispanics was available, the prevalence of anemia we obtained (41.5% overall; 44.4% in women and 40.0% in men) was substantially higher. For example, in the Anemia Prevalence Study, a large multicenter study carried out in HIV-positive people in the United States, the overall prevalence of anemia in 1,529 Hispanics was 31.3% (26.1% for women and 33.0% for men) [8]. Similarly, the Women’s Interagency HIV Study included 475 Hispanic patients, and reported an anemia prevalence of 24.8% [6]. Finally, in a cross-sectional study of HIV-infected patients of Mexican descent (n = 63), the overall prevalence of anemia was 20.3% [13].

Many reasons may explain why our cohort has an elevated prevalence of anemia. First of all, our study included a high number of patients with advanced immunological deterioration (41.8% of patients had a CD4 cell count <200 cells/μL), which has been associated to the development of anemia during HIV infection [17,22]. Also, our institution receives a large number of care referrals from patients of low socioeconomic status, as demonstrated by the 75.0% of patients who reported being unemployed. This could lead to a sicker population with delayed health seeking activities [28], and it is well known that at a later stage of HIV disease the development of comorbidities, such as anemia, is more common [29]. We do not believe that the higher anemia prevalence in our population is related to a different standard in defining anemia, since the definition we used is actually more conservative than in other published works. All three cited studies with Hispanic participants had the same hemoglobin cutoff than us for women (<12 g/dL), but in the Anemia Prevalence Study and Mata-Marín and colleagues’ study, a cut-point of <14 g/dL for men (1 g/dL higher) was used. If employing this value in our study, the prevalence of anemia in men would have been higher at 57.9%, with an increased overall prevalence of anemia of 53.5%.

Significant differences in the prevalence of anemia by socio-demographic, clinical and immunological factors were observed in this study. The finding that anemia is more prevalent in older patients and those with lower BMI, lower CD4 cell count, higher HIV viral load and clinical AIDS has been previously documented [6,8,10,14]. When evaluating these and other factors in the multivariable model, unemployment, CD4 count <200 cells/μL, HIV viral load >100,000 copies/mL, presence of clinical AIDS and white blood cell count <4,000 cells/μL, were independently associated with augmented odds of anemia. Conversely, overweight and obese BMI’s, were found to be associated with reduced odds of anemia. With the exception of unemployment, all these factors have been associated to anemia in earlier studies [6-10,12,13,19,20].

To our knowledge, the relationship of unemployment and anemia in HIV-infected patients has not been reported before. Nonetheless, the design of this study does not allow us to have a clear understanding of the finding. Employment status is used as an indicator of socioeconomic position, and has been shown that unemployed people are less likely to receive an adequate medical care [28]. HIV-infected patients under these conditions may not be able to prevent the progression of HIV, which could contribute to a deterioration of their health and the occurrence of complications, such as anemia. On the other hand, it can be argued that the presence of fatigue and weakness associated to anemia, which affect the quality of life and physical functioning of patients [3,22,30], could also impede sustained employment. Thus, future research efforts should be directed to evaluate this association.

In agreement with several studies, we found a relationship between lower levels of hemoglobin and higher one-year mortality rates. A multicenter study conducted in Europe revealed that the proportion of dead patients after a year of follow-up was 40.8% for patients with severe anemia (<8 g/dL), 15.9% for patients with mild anemia (8–12 g/dL in women and 8–14 g/dL in men) and 3.1% for patients without anemia (>12 g/dL for women and >14 g/dL for men) [4]. Likewise, in a study of HIV-infected patients from Tanzania, the one-year mortality estimates were: 55.2% in patients with severe anemia (<8 g/dL), 37.6% in patients with moderate anemia (8–9.9 g/dL), 20.0% in patients with mild anemia (10–11.9 g/dL in women and 10–12.9 g/dL in men), and 3.7% in patients without anemia (≥12 g/dL for women and ≥13 g/dL for men) [27].

The observation of anemia being a predictor of one-year mortality in our cohort was confirmed when we analyzed possible confounders in the proportional hazards model. Other studies have also documented this independent association [4,31]. In the EuroSIDA study, a decrease in 1 g/dL of hemoglobin level augmented the hazard of death (HR = 1.57; 95% CI: 1.41-1.75), after controlling for demographic variables, antiretroviral treatment, AIDS, CD4 count and viral load [4]. In addition, Alemu and colleagues concluded that anemia was a predictor of mortality in a group of patients living in Ethiopia, after adjusting for treatment, WHO clinical stage and body weight [31].

In this study we found that anemia was the strongest predictor of one-year mortality and that a higher degree of anemia was related to a greater risk of dying. Similarly, in a cohort of 2,348 HIV-positive patients from the United States, severity grades of anemia were associated with an increased mortality risk, after controlling for CD4 cell count, age, antiretroviral treatment and development of opportunistic infections [21]. Despite these results, we consider the relationship between anemia and one-year mortality in our population is not necessarily causal. There are elements that could be mediating in the mentioned association and were not included in this study. For example, information regarding blood transfusions, a common therapeutic practice for the temporary correction of anemia, was not addressed. Studies have associated this treatment with a worse prognosis in HIV-infected patients, since it may have an immunosuppressive effect on their already weakened immune system [17,32]. Therefore, we believe that instead of causing deaths directly, anemia might be an indicator of a compendium of comorbid systemic states [20], which ultimately contribute to these patients’ demise.

Our data provides additional evidence on the role of anemia during HIV infection and also reveals its great impact in this cohort of Hispanic subjects. Nevertheless, limitations of this study should be mentioned. Firstly, as variables were compiled at the same time, we were unable to establish that factors associated with anemia were present before the disorder, and are either risk or protective factors. In addition, all data we analyzed (except mortality information) was obtained at the time of study entry. Not evaluating clinical and laboratory information after baseline impeded us from having a more precise scenario of the relationship between many variables that change over time (i.e., CD4 cell count, HIV viral load, BMI, treatment, hemoglobin level) and survival. Finally, as the sample for this study was conveniently drawn, we cannot generalize our results to all HIV-infected patients in Puerto Rico.

## Conclusions

The presence of anemia at enrollment was relevant in this cohort of HIV-infected Hispanics, and it was associated with several adverse prognostic features of the HIV infection and unemployment. Most importantly, having anemia at baseline was independently associated with an increased mortality risk at one year. Future steps should focus on integrating the patients’ follow-up information to evaluate whether recovering from anemia is associated with longer survival, as has been described [19,21]. Further investigations are also needed to identify the types of anemia affecting these patients, which would bring valuable information on the causes of this abnormality, and will help to delineate strategies in order to deal with this threatening comorbidity in this particular group.

## Abbreviations

RBC: Red blood cell; BMI: Body mass index; UCC: Universidad Central del Caribe; Hb: Hemoglobin level; OR: Odds ratio; HR: Hazards ratio; CI: Confidence interval; ART: Antiretroviral treatment.

## Competing interests

The authors declare that they have no competing interests.

## Authors’ contributions

EJS performed the statistical analysis and drafted the manuscript. AMM and DMF contributed with recommendations on study design and analysis and also edited the draft. YRC helped in study conceptualization and edited the draft. RFH conceived the study and participated in results interpretation and draft editing. All authors revised and approved the final version of the manuscript.
